# Emerging roles of Sox6 in the renal and cardiovascular system

**DOI:** 10.14814/phy2.14604

**Published:** 2020-11-23

**Authors:** Mohammad Saleem, Pierina Barturen‐Larrea, Jose A. Gomez

**Affiliations:** ^1^ Department of Medicine / Clinical Pharmacology Division Vanderbilt University Medical Center Nashville TN USA

## Abstract

The function of Sex‐determining Region Y (SRY)‐related high‐mobility‐group box (Sox) family of transcription factors in cell fate decisions during embryonic development are well‐established. Accumulating evidence indicates that the Sox family of transcription factors are fundamental in adult tissue homeostasis, regeneration, and physiology. The SoxD subfamily of genes are expressed in various cell types of different organs during embryogenesis and adulthood and have been involved in cell‐fate determination, cellular proliferation and survival, differentiation, and terminal maturation in a number of cell lineages. The dysregulation in the function of SoxD proteins (i.e. Sox5, Sox6, Sox13, and Sox23) have been implicated in different disease conditions such as chondrodysplasia, cancer, diabetes, hypertension, autoimmune diseases, osteoarthritis among others. In this minireview, we present recent developments related to the transcription factor Sox6, which is involved in a number of diseases such as diabetic nephropathy, adipogenesis, cardiomyopathy, inflammatory bowel disease, and cancer. Sox6 has been implicated in the regulation of renin expression and JG cell recruitment in mice during sodium depletion and dehydration. We provide a current perspective of Sox6 research developments in last five years, and the implications of Sox6 functions in cardiovascular physiology and disease conditions.

## Graphical abstract

The function of Sex‐determining Region Y (SRY)‐related high‐mobility‐group box (Sox) family of transcription factors in cell fate decisions during embryonic development are well‐established. The SoxD subfamily of genes are expressed in various cell types of different organs during embryogenesis and adulthood and dysregulation in the function of SoxD proteins (i.e. Sox5, Sox6, Sox13, and Sox23) have been implicated in different disease conditions. In this minireview, we present recent developments related to the transcription factor Sox6, which is involved in a number of diseases such as diabetic nephropathy, adipogenesis, cardiomyopathy, inflammatory bowel disease, and cancer. We provide a current perspective of Sox6 research developments in last five years, and the implications of Sox6 functions in cardiovascular physiology and disease conditions.

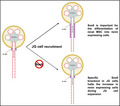

## 1 INTRODUCTION

The Sex‐determining Region Y (SRY)‐related high‐mobility‐group box (Sox) gene family is comprised of 20 genes in vertebrates and 12 genes in invertebrates (Wegner, 2010). The Sox family of transcription factors contain the high‐mobility‐group (HMG) box DNA‐binding domain. Though there are discrepancies, the Sox genes classification include 10 groups A to J (Wegner, 2010).

The SoxD subfamily amino acid sequence does not contain transactivation or trans‐repression domains. However, they have function in transcriptional activation and repression (Han & Lefebvre, 2008; Lefebvre, 2010). Detailed information about the structure, expression and regulation, biological function, and medical relevance of SoxD subfamily has been reviewed elsewhere (Lefebvre, 2010). The SoxD subfamily includes the transcription factors Sox5, Sox6, Sox13, and Sox23. Several studies demonstrated that Sox6 contains DNA binding domain and binds to the minor groove of DNA (Hagiwara, 2011; Han & Lefebvre, 2008). Furthermore, Sox6 function in gene regulation is multifaceted and as such it can function by directly binding to DNA, or interacting with cofactors, or micro‐RNAs (miRNAs; Iguchi et al., 2005; Leow et al., 2016; Li et al., 2019; Yi et al., 2006; Yousefzadeh et al., 2017). The ability of Sox6 to interact with different proteins at different places and times, makes it an intricate protein with roles in multiple processes (Hagiwara, 2011). Sox6 has been implicated in diabetic nephropathy (Jiang et al., 2020; Qi et al., 2019), adipogenesis (Leow et al., 2016), cardiomyopathy (Yousefzadeh et al., 2017), inflammatory bowel disease (Kinchen et al., 2018), and cancer (Liang et al., 2019). Several reviews have been published emphasizing the functional importance of Sox6 in embryonic development, mesenchyme cells differentiation (chondrocyte and skeletal muscle differentiation), central nervous system development (oligodendrocyte and neural differentiation), skeletogenesis (osteoblast differentiation), erythropoiesis, and cancer (Hagiwara, 2011; Ji & Kim, 2016; Lefebvre, 2010; Liang et al., 2019; Wegner, 2010). However, there are no reviews yet of any kind which specifically focus on the functional importance of Sox6 in the renal and cardiovascular systems. In this review, we focus on the function of Sox6 in cardiovascular diseases, hypertension, and diabetes and related conditions.

## 2 SOXD SUBFAMILY: STRUCTURE AND FUNCTION IN CELL FATE AND PHYSIOLOGY

**FIGURE 1:**
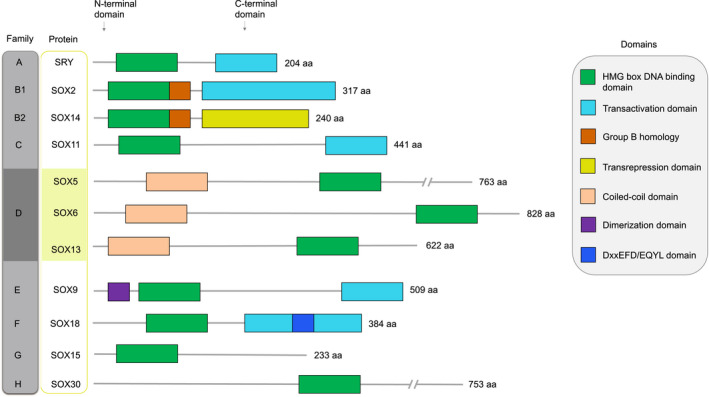
**A pictorial depiction of the distinct domains of protein structure of the human SOX family members.** Representative protein structure of the Sox family members from A to H groups. This classification is based on gene structure and function in embryonic developmental processes, phylogenetic studies, and proteins structures (Grimm et al., 2019). The HMG (high mobility group) box, the highly conserved and characteristic feature of Sox family members, is depicted alongside other key domains such as trans‐activation/repression domain (absent in Sox D subfamily), and coiled‐coil domain (present only in Sox D subfamily). The coiled‐coil domain features LZ and Q‐boxes which help Sox D family members to interact among them and with other proteins, cofactors, and miRNA (Han & Lefebvre, 2008; Iguchi et al., 2005). At the top, from left to right: Sox families, representative members, N‐terminal, C‐terminal are denoted. The number of amino acids (aa) for each protein are shown at the right side. Various domains of representative protein of each subfamily are mentioned in the box at the right

The SoxD subfamily has been implicated in the transcriptional regulation of developmental processes, including embryonic development, cartilage formation, hematopoiesis, and nerve growth (Connor et al., 1995; Lefebvre, 2010). The SoxD family genes were cloned in the 90s when the scientists were searching for Sry‐related genes (Connor et al., 1995; Lefebvre, 2010). Similar to other Sox family members, Sox D subfamily members contain an HMG box in the C‐terminus that can bind to DNA. However, SoxD family structure differs from the other Sox proteins in the N‐terminus sequence that contains a leucine zipper (LZ) and glutamine‐rich (Q‐box) domains (Figure 1; Hagiwara, 2011; Lefebvre, 2010). In particular, Sox6 features the HMG box functional domain in the C‐terminal, and two coiled‐coil functional domain in the N‐terminal where the LZ and Q‐boxes are located (Connor et al., 1995; Lefebvre, 2010). The coiled‐coil domain is the characteristic feature of Sox D subfamily which is absent in other Sox family members. Leucine zipper helps to make homo‐ or ‐heterodimers with other Sox family members, and proteins. For example, Sox6 via Q‐box and LZ interacts with β‐catenin and Sox5 and makes homo‐ and ‐heterodimers with other Sox proteins (Han & Lefebvre, 2008; Iguchi et al., 2005). The coiled‐coil domain contains the LZ and Q‐boxes, and most of the interactions between Sox6 and other proteins are mainly performed in the coiled‐coil domain (Han & Lefebvre, 2008; Lefebvre, 2010). The in vitro electrophoretic mobility shift assay (EMSA) shows that SoxD family transcription factors preferentially bind with DNA sequences featuring an AACAAT motif (Connor et al., 1995). In vivo and in vitro studies revealed that the proteins efficiently bind to the site containing up to two mismatches in the favored sites. Also, the proteins possess slight inclination for the relative orientation of the paired sites and for the length of the intervening sequence, from 0 to at least 19 bp (Han & Lefebvre, 2008). Due to such flexibility, putative binding sites for SoxD family proteins could be found in any DNA regulatory or promoter region. It is required that robust experiments must be designed and carried out to establish the actual sites for SoxD proteins in vivo.

Sox6 is expressed in different cells, such as neurons, oligodendrocytes, chondrocytes, cardiomyocytes, erythrocytes, skeletal myoblasts, spermatids, pancreatic b‐cells, kidney, and liver cells (Hagiwara et al., 2000; Shi et al., 2019; Yousefzadeh et al., 2017). Sox6 has crucial functions in the developing mouse embryo, in the differentiation of skeletal and cardiac myocytes (Shi et al., 2019; Yousefzadeh et al., 2017), skeletal/cartilage tissues (Lefebvre, 2019), central nervous system (CNS) (Ji & Kim, 2016), melanocytes (Stolt et al., 2008), erythroid, Th17 (Tanaka et al., 2014), and hair follicle, and inner ear (Lefebvre, 2010). Interestingly, Sox6 is also expressed in adult mice, suggesting roles in the maintenance of adult tissues with some still unexplored.

## 3 Sox6 AND CARDIOVASCULAR DISEASES

Studies in early 2000 reported that systemic deletion of the Sox6 gene in mice results in death two weeks after birth due to progressive atrioventricular heart block and ultrastructural changes in both cardiac and skeletal muscles (Hagiwara et al., 2000). These observations indicate that function of Sox6 is critical in development and preservation of both cardiac and skeletal muscles (Hagiwara et al., 2000). The postnatal heart experiences remarkable transformation at the physiological, cellular, and molecular levels such as changes in the cardiomyocyte growth, and shift from hyperplasia to hypertrophy, multinucleation and polyploidation, to attain the complex physiological activity of the adult heart (Li et al., 1996). A recent report showed that Trbp (Tarbp2), an RNA‐binding protein, controls the expression of miR‐208a that repress the expression of Sox6 to maintain normal cardiac function. Sox6 is the key protein that maintains the balance of gene expression for slow and fast twitch protein isoforms. They showed cardiac specific Trbp KO mice express lower miR‐208a and overexpress Sox6 that result into progressive cardiomyopathy and lethal heart failure (Ding et al., 2015). In another report, Chung‐Il An et al. 2016, the authors identified Sox6 as key transcription factors suppressing cell proliferation and fine‐tuning fetal gene expressions during postnatal heart development to culminate in the complex functional heart (An et al., 2016). Yousefzadeh et al. (2017) reported that fetal hypothyroidism decreases cardiac performance as a result of decreased ratio of α‐MHC:β‐MHC expressions in adult rats. The decreased expression of α‐MHC:β‐MHC ratio is attributed to the imbalances in cardiac myomiR network (increased expression of miR‐499, 208b, decreased expression of miR‐208a) in fetal heart. These changes in myomiR network decrease the expression of Sox6, Thrap1, and Purβ resulting in the increased expression of β‐MHC which is responsible for lower cardiac efficiency (Yousefzadeh et al., 2017). Another study showed that miR‐499 inhibits hypoxia/reoxygenation (H/R) induced apoptosis in cardiomyocytes by downregulating Sox6 expression (Shi et al., 2019). Similarly, Zhang et al. 2019 showed that Sox6 is the target gene for miR‐208b and miR‐499‐5p. This study showed that butyrate diet increases the expression of miR‐208b and miR‐499‐5p that decrease the expression of Sox6 and Sp3 proteins expression and thereby increases the synthesis of slow‐twitch myofiber and mitochondrial biogenesis and increase the meat quality in pigs (Zhang et al., 2019). A study by Lin et al. (2018) revealed that Sox6 expression is positively associated with copy number variations (CNVs). This study further revealed that expression of Sox6 is highly expressed during the skeletal muscle cell differentiation in chickens (Lin et al., 2018). Vestigial‐like factor 2 (VgII‐2) is predominantly expressed in skeletal muscle and plays a role in muscle‐specific gene expression. A study in VgII‐2 KO mice showed that these mice exhibit lower expression of miR‐208b and upregulation of its target genes Sox6, Sp3, and Pur‐β. This study concluded that miR‐208b by targeting Sox6, Sp3, and Pur‐β plays important function in skeletal muscle development in mice (Honda et al., 2017). Mesenchymal stem cells derived exosomes show protective effect against acute myocardial infarction (AMI) and promote cardiac regeneration and angiogenesis in in vivo and in vitro studies (Huang et al., 2020; Ma et al., 2017; Wang et al., 2018). Huang et al. 2020 showed that human umbilical cord mesenchymal stem cells (hucMSCs) cells releases exosomes that contain miR‐19a. miR‐19a is protective against AMI in rats and hypoxia in H9C2 cells. This study further revealed that miR‐19a inhibits Sox6 gene to protect H9C2 cells against hypoxic injury (Huang et al., 2020).

Sepsis‐induced cardiac apoptosis has been established as a pathogenic factor implicated in myocardial dysfunction. Lipopolysachharide (LPS) is considered to be one of the key mediators in this pathological condition. Overexpression of Sox6 and PDCD4 (programmed cell death 4) genes by LPS inhibit miRNA‐499 that results into enhanced apoptosis in cardiomyocytes (Jia et al., 2016). Muscle‐specific knockout of Sox6 revealed its function as a transcriptional repressor in slow fiber specific genes during both prenatal and postnatal muscle development in cardiac and skeletal muscle and is indispensable for the optimal performance and health of the cardiac and skeletal muscles (An et al., 2011; Quiat et al., 2011). The β‐thalassemia is an autosomal recessive disorder, characterized by the deficiency of β‐chain synthesis. A dysregulated synthesis of β‐globin results in an imbalance in the α‐/β‐chain ratio leading to red blood cells (RBCs) lysis (Lidonnici & Ferrari, 2018). During β‐thalassemia, reactivation of the fetal γ‐globin decreases the abundance of α‐globin chains, resulting in lowered RBC destruction (Dreuzy et al., 2016; Sankaran et al., 2010). The transcription factor Sox6 is a pivotal gene in switching the γ to β‐globin gene. Shariati et al. (2018), reported that disruption of Sox6 binding domain results in the reactivation of γ globin expression. This study concluded that silencing Sox6 may become therapeutic strategy for β‐thalassemia treatment (Modares Sadeghi et al., 2018; Shariati et al., 2018). Another study showed the functional importance of a number of miRNAs and their target Sox6 in hereditary persistence of fetal hemoglobin deletion type‐2 (HPFH‐2) and Sicilian‐δβ‐thalassemia diseases (Fornari et al., 2017).

**FIGURE 2:**
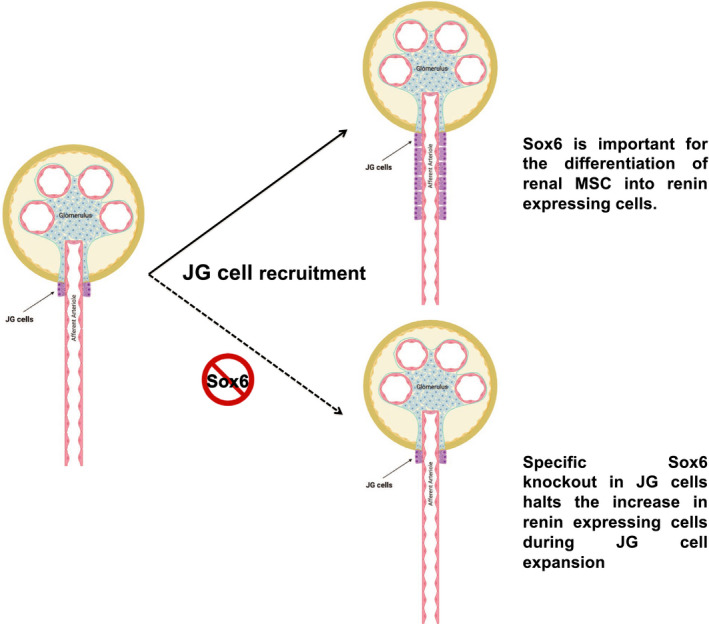
**The role of Sox6 in various organ systems and implication in diabetes and cardiomyopathy.** Overexpression/activation of Sox6 by adipogenic stimuli increases the expression of CEBPα and PPARγ leading to adipogenesis. Sox6 increases the expression of MEST gene that in turn reduces WNT signaling. Sox6 binds and degrades the β‐catenin that further leads to reduced WNT signaling causing the adipocyte multiplication (Leow et al., 2016). The miR‐802 increases Sox6 expression by inhibiting the p‐CREB expression leading to reduced insulin secretion (Zhang et al., 2020). Lower expression of all the miRs with asterisk (*) results into the overexpression of Sox6 causing the reduction in the number of insulins producing cells and synthesis/secretion of insulin. For detail, please read (Bai et al., 1859; Bai et al., 2017; Li et al., 2019; Qi et al., 2019). Sox6 by interacting with PDX1 and reducing the acetylation of histone 3 and 4 in the chromatin of insulin promoter that reduces the binding of PDX1 causes reduced insulin secretion (Iguchi et al., 2005). Trbp by inhibiting miR‐208a, and miR‐499 increases the expression of Sox6 that results into lower expression of slow‐muscle twitch fiber, these fibers are indispensable for cardiac muscle efficiency in mice (Ding et al., 2015). Increased expression of miR‐499, 208b, and decreased expression of miR‐208a inhibit Sox6 expression causing decreased ratio of α‐MHC:β‐MHC leading to lower cardiac muscle efficiency in rats (Yousefzadeh et al., 2017). *Abbreviations:* PPARγ, peroxisome proliferator activated receptor gamma; MEST, mesoderm specific transcript; miR, micro‐RNA; p‐CREB, phospho‐cAMP response element‐binding protein; PDX1, pancreatic‐duodenal homeobox factor‐1; Trbp, transactivation response element RNA‐binding protein; MHC, myosin heavy chain

Published reports show the functions of Sox6 are imperative in the optimum functioning of cardiac and skeletal muscles during both embryonic development and adulthood. Sox6 is the target gene for a number of miRNAs which are functionally important in regulating a number of cardiac and skeletal muscles’ functions. Overexpression of Sox6 has been implicated in cardiac muscle apoptosis, AMI, and β‐thalassemia. Over the last two decades a fair amount of research has been reported in the field, however, further studies are needed to strengthen the function of Sox6 in cardiovascular pathophysiology (Figure 2).

## 4 Sox6 AND DIABETES

Diabetes is one of the leading causes of premature deaths in the world. United States ranks third in diabetes cases after India and China, the main epicenters of diabetes (Cho et al., 2018). The pathophysiology of the disease is multifactorial and complex. Several studies have linked obesity with an increased risk of diabetes (Leow et al., 2016; Sloten et al., 2011). Diabetes is one of the main contributors to cardiovascular and kidney complications resulting in morbidity and mortality. Leow et al. 2016, reported that Sox6 regulates adipogenesis in vertebrates by inducing adipogenic regulators such as PPARγ (peroxisome proliferator‐activated receptor), C/EBPα, and MEST (mesoderm specific transcript). They also found that Sox6 promotes adipogenesis by inhibiting the WNT/β‐catenin pathway in adipocytes (Leow et al., 2016). Several studies report that microRNAs (miRNAs) control pancreatic development, beta‐cell differentiation, and insulin secretion (Bai et al., 2017). Bai et al. 2016, examined the profile expression of microRNAs in nestin‐positive umbilical cord‐derived mesenchymal stem cells (N‐UCMSCs) and nestin‐positive pancreatic mesenchymal stem cells using deep sequencing. They found that the inhibition of Sox6, mtpn, bhlhe22, and ccnd1 leads to miR‐375, and miR‐26a overexpression in N‐UCMSCs. Therefore, promoting the differentiation of N‐UCMSCs into insulin‐producing cells (Bai et al., 2017). They also studied the role of miRNA‐21 and determined that miRNA‐21 by inhibiting Sox6 increases insulin‐producing cells formation (Bai et al., 2016). To further investigate the involvement of Sox6 and miRNAs in diabetes regulation, a study revealed that miRNA‐96 is upregulated under the pathological condition of type‐2 diabetes mellitus (T2DM). miRNA‐96 KO mice exhibit pancreatic β‐cell dysfunction, increased fasting blood glucose during T2DM. Overexpression of miRNA‐96 by inhibiting Sox6 and Foxo1 increases the proliferative ability and inhibits apoptosis in mouse insulinoma (MIN6) cells (Qi et al., 2019).

Pioneering studies showing the functional role of Sox6 in insulin regulation revealed that insulin promoter contains the binding site for Sox6 transcription factor and Sox6 by inhibiting pancreatic‐duodenal homeobox factor‐1 (PDX1) reduces insulin secretion (Iguchi et al., 2005). The miRNA‐802 is highly upregulated in islets of the pancreas of obese mouse models. Using knockout and knock‐in approaches Zhang et al. (2020), showed that miRNA‐802 regulates synthesis of insulin. In this study, they revealed that increased expression of miRNA‐802 downregulates phosphorylation of CREB, that results into overexpression of Sox6 and dysregulation of insulin secretion and synthesis (Zhang et al., 2020). Another study shows the involvement of miRNA in insulin and diabetes regulation. Li et al. (2019), investigated the role of miRNA‐223 in maintaining functional mass of pancreatic β‐cells and insulin secretion. They used miRNA‐223 KO mice and found that these mice exhibit increased expression of both Sox6 and Foxo1 (forkhead box O1) genes, and impaired glucose tolerance and insulin resistance as a result of the suppression of β‐cells proliferation and insulin secretion. Using different approaches, they showed that miRNA‐223 exhibits its function by inhibiting Sox6 and Foxo1 pathways (Li et al., 2019). Type 1 and/or T2DM are one of the main contributors of microvascular complications during diabetic kidney disease (DKD) which is the main cause of end‐stage renal disease (ESRD; Lu et al., 2017). Using a DKD model, and mouse renal mesangial cells (MCs), Jiang and colleagues studied the miRNA‐342 mediated regulation of Sox6 and impacts on interstitial fibrosis during diabetic nephropathy (Zhang et al., 2020). They showed that miRNA‐342 binds to the 3’‐UTR of Sox6 and inhibits its expression. Results from the study revealed that the inhibition of Sox6 by miRNA‐342 diminished the progression of DKD (Jiang et al., 2020). Interestingly, Pleskovič et al investigated the association of Sox6 gene polymorphism (rs16933090) with all the subclinical markers of carotid atherosclerosis such as the number of affected segments of carotid arteries, plaque thickness, and carotid intima media thickness (CIMT), in T2DM patients. The results from the study suggest that polymorphism of Sox6 gene (rs16933090) in T2DM patients precipitates subclinical markers of atherosclerosis (Pleskovic et al., 2016).

Collectively, Sox6 is shown to play an important function in adipogenesis by inducing PPARγ and inhibiting WNT/β‐catenin pathways. Overexpression of Sox6 is implicated in inhibiting insulin synthesis, and insulin producing cells, and reducing β‐cell mass and insulin secretion. So, Sox6 could be a potential therapeutic target to design better drug to treat diabetes and associated complications (Figure 2).

## 5 Sox6 IN HUMAN HYPERTENSION

Blood pressure (BP) is a multifactorial disease and a cardinal risk factor for cardiovascular diseases. In addition to the function of Sox6 in the development, and cardiovascular diseases, Sox6 has also been associated with human hypertension. Johnson et al. 2011, in discovery analysis genotyped 49,452 single‐nucleotide polymorphisms (SNPs) in 25,118 individuals and identified SNPs in Sox6 loci that were highly associated with blood pressure. By performing follow‐up analysis in additional 59,349 individuals, they confirmed the associations of Sox6 SNPs in one of the blood pressure traits such as DB, SBP, MAP, PP, and hypertension (Johnson et al., 2011). Using gene‐centric array approach, Ganesh et al. 2013, in discovery analysis genotyped more than fifty thousand SNPs in approximately 2,100 candidate genes for hypertension and cardiovascular disease in 61,619 individuals of European ancestry from cohort studies in the Europe and USA. Discovery analysis associates Sox6 SNPs with systolic blood pressure. The role of Sox6 SNPs in systolic blood pressure was confirmed by performing replication analysis in 65,866 additional individuals (Ganesh et al., 2013). Franceschini et al 2013, performed meta‐analysis in 19 discovery cohorts (*n* = 29,378 subjects) from the Continental Origins and Genetic Epidemiology Network (COGENT) GWAS of African‐Ancestry (AA) samples for BP trait and identified a novel SNP in a known BP locus for Sox6 (Franceschini et al., 2013). Transethnic meta‐analysis by combining discovery AA samples and replication samples from additional AA samples (*n* = 10,386), European ancestry (EA) (*n* = 69,395), and East Asian ancestry (*n* = 19,601) also associates Sox6 SNP with blood pressure and infers that Sox6 SNPs are associated with blood pressure independent of ethnicities (Franceschini et al., 2013). The association of Sox6 SNPs with blood pressure regulation were replicated in Chinese population (*n* = 80,962), again inferring that Sox6 SNPs are associated with blood pressure traits across ethnicities (Lu et al., 2015).

GWAS studies associate Sox6 SNPs with human hypertension across the ethnicities and imply great potential of functional studies for Sox6 in hypertensive animal models to discern the Sox6 function and establish molecular pathway/s in hypertension.

## 6 Sox6 AND RENIN REGULATION IN THE KIDNEY

To investigate the functional role of Sox6 in renal renin regulation, microarray in resident mesenchymal stromal cells (MSCs) and juxtaglomerular (JG) cells was performed (Saleem et al., 2020).

**FIGURE 3:**
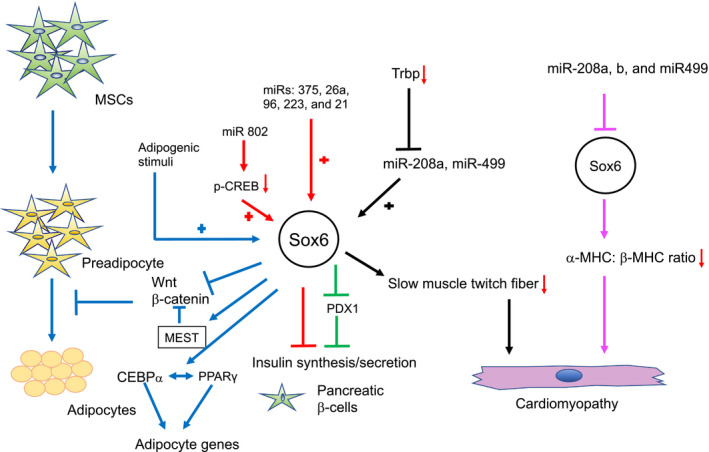
**The increase in renin expressing cells during JG cell recruitment is inhibited by specific Sox6 ablation in renin expressing cells.** Schematic overview of juxtaglomerular (JG) cells recruitment (indicated by arrows) that occurs in the adult kidney

This study revealed that Sox6 expression increases in JG cells in the adult kidney. Knock down of Sox6 gene halted the differentiation of MSCs into renin expressing cells in in vitro studies. Systemic Sox6 knockout mice do not survive after 14 days of birth due to abnormalities in cardiac and skeletal muscles development. We and others have shown that renin promoter possesses the binding site for Sox6 (Modares Sadeghi et al., 2018), and along with other transcription modulators regulate renin expression (Martinez et al., 2018). Using a loss of function mouse model, in which Sox6 is specifically knockout in renin expressing cells, Sox6 was shown to regulate renin expression and JG recruitment in response to sodium depletion and dehydration (Figure 3; Saleem et al., 2020). These studies show that Sox6 is one of the main contributors of renin regulation. Renin is the rate‐limiting enzyme in renin angiotensin aldosterone system (RAAS). RAAS is implicated in hypertension, oxidative stress, and other cardiovascular diseases (Ken & Hackett, 1991). Sox6, being the regulator of renin, has great potential to control RAAS and could be a potential therapeutic target in RAAS associated diseases. Further studies are warranted to unravel the molecular mechanism involved in Sox6 mediated renin regulation.

## 7 PERSPECTIVE/CONCLUSION

The compiled information presented here highlights the functional importance of the transcription factor Sox6 in cardiomyopathy, diabetes, and in renin regulation in adult tissues. GWAS studies associate Sox6 SNPs with human hypertension across ethnicities (Johnson et al., 2011) and imply great potential of functional studies for Sox6 in hypertensive animal models to discern the Sox6 function and establish molecular pathway in hypertension (Saleem et al., 2020). The investigations in the field of cardiomyopathy and diabetes revealed that Sox6 is primarily regulated by various types of miRNAs (An et al., 2016; Bai et al., 2016). Sox6 is a target gene for the miRNAs which are functionally important in regulating a number of cardiac and skeletal muscles functions. Overexpression of Sox6 has been implicated in cardiac muscle apoptosis, AMI, and β‐thalassemia. Moreover, overexpression of Sox6 has been associated with decreased β‐cell mass and insulin secretion, and increased apoptosis in insulin‐producing MIN6 cell (Qi et al., 2019). A number of studies revealed that Sox6 by interacting with other transcription factors, cofactors, or miRNA can enhance or repress the target gene expression depending on spatial and temporal situations during physio‐ and pathophysiological processes. Although, a number of studies already revealed the importance of the transcription factor in various physio‐ and pathophysiological processes, Sox6 studies are still in early stage in adult animals and warrants further investigation. Due to the versatile nature, future studies have the great potential to reveal undiscovered functions of Sox6 gene and the protein in both physio‐ and pathophysiological processes.

## CONFLICT OF INTEREST

No conflicts of interest, financial or otherwise, are declared by the authors.

## AUTHOR CONTRIBUTIONS

M.S., and P.B.L., wrote the manuscript, figures, and table drafts. J.A.G., edited the paper, figures, and table and approved the final version.

## ETHICAL STATEMENT

Dr. Gomez is an Assistant Professor at Vanderbilt University Medical Center. Dr. Gomez laboratory is funded by an NHLBI Research Scientist Development Grant (1K01HL135461), and in part by discretionary research funds from the Vanderbilt University Medical Center.

8

**Table 1:** The role of Sox6 in various organ system involving various molecular pathways/genes

**Organ systems**	**Affected Cell types**	**Sox6 expression**	**Pathways/genes involved**	**Function/Disease**	**References**
Heart	Cardiomyocytes	Overexpression	Trbp‐miR−208a	Apoptosis Reduced heart efficiency	Ding et al. (2015); Jia et al. (2016); Yousefzadeh et al. (2017)
Skeletal muscle	Myocytes	Overexpression	Vestigial‐like factors, Vgll2	Reduced muscle mass and efficiency	Honda et al. (2017)
Blood	RBCs	Overexpression	the γ to β‐globin gene	Thalassemia	Modares Sadeghi et al. (2018); Shariati et al. (2018)
Kidney	JG cells	Overexpression	Renin gene	Renin overexpression	Saleem et al. (2020)
	Mesangial cells	Overexpression	Not Known yet	Interstitial fibrosis Diabetic nephropathy	Jiang et al. (2020); Zhang et al. (2020)
Whole body	All cell types	Complete deletion		Death due to arterioventricular heart block and ultrastructural changes in both cardiac and skeletal muscles	Hagiwara et al. (2000)
Adipose tissue	Adipocyte	Overexpression	PPARγ C/EBPα, MEST WNT/β‐catenin	Adipogenesis	Leow et al. (2016)
Pancreas	Islets (β‐cells)	Overexpression	PDX1	Reduced insulin secretion, diabetes	Bai et al. (2017); Bai et al. (1859); Iguchi et al. (2007)
